# Latent preference for red ornamentation drives interspecific mating in nascent jumping spider species (*Habronattus americanus group, F. Salticidae*)

**DOI:** 10.1098/rspb.2024.2754

**Published:** 2025-08-20

**Authors:** Lin Yan, Noah Joon Huh, Daniel Ibañez IV, Malcolm F. Rosenthal, Marshal Hedin, Damian O. Elias

**Affiliations:** ^1^ Department of Environmental Science, Policy and Management, University of California Berkeley, Berkeley, CA 94720, USA; ^2^ Biodiversity Institute, University of Kansas, Lawrence, KS 66045, USA; ^3^ Columbia University, New York, NY 10027, USA; ^4^ Department of Biology, San Diego State University, San Diego, CA 92182, USA

**Keywords:** mate choice, speciation dynamics, latent preference, heterospecific interactions, Habronattus, extinction

## Abstract

Heterospecific interactions between nascent species offer insights into how sexual selection shapes novel traits, illuminating patterns in species interactions and diversification. We tested female preferences between two recently diverged, allopatric species of jumping spiders: *Habronattus americanus* PLC, with red-coloured males performing short multimodal displays, and *Habronattus sansoni* CC, with brown-coloured males performing long multimodal displays. Mate choice experiments showed that females of both species preferred *H. americanus* PLC males. To examine the role of red coloration, we manipulated male coloration in both species. Results indicated that red-painted *H. sansoni* CC males experienced an increase in mating success, whereas brown-painted *H. americanus *PLC males did not show reduced success. Our study suggests that (i) strong latent female preferences can drive unidirectional introgression across species boundaries, potentially leading to genomic homogenization; (ii) latent preferences may override preferences for existing traits; and (iii) the geographical distribution of colour morphs is consistent with a hypothesis of strong latent preferences across populations. Overall, our study demonstrates the role that mating interactions can play in speciation dynamics.

## 1. Introduction

The prevalence of introgression across the tree of life suggests that hybridization is a key feature in speciation [1]. Speciation does not necessarily follow a simple, linear path where the evolution of nascent species is followed by the emergence of strong barriers to gene flow and subsequent complete reproductive isolation. Instead, speciation is a complex reticulate process where factors associated with hybridization (e.g. hybrid fitness, frequency of hybridization and niche availability for hybrids) can increase, stabilize or erode divergence at any stage of the speciation process [2–5]. To understand these complexities, it is necessary to examine the factors that promote hybridization between individuals of divergent populations.

Mate preferences are thought to be the primary pre-zygotic barrier preventing hybridization between closely related species, as intrinsic post-zygotic incompatibilities are believed to evolve later in species that recently diverged [6,7]. Selection against hybrids with reduced fitness can drive mate preferences to filter out inappropriate courters (i.e. heterospecifics), thus reinforcing assortative mating and creating barriers to hybridization. This, however, is not always the case. In some instances, mate preferences may increase interspecific gene flow, eroding reproductive barriers [8–11]. For example in manakins (family Pipridae), mating preferences for plumage traits were shown to drive asymmetric trait introgression in a hybrid zone [12]. These and other studies have suggested that asymmetric gene and trait introgression can blur phenotypic differences across species boundaries and potentially drive genomic homogenization (variously referred to as species collapse [13], ‘despeciation’ [14] or ‘speciation in reverse’ [15]). Importantly, while courter signals and chooser preferences (including associated sensory systems) are generally thought to be selected to be an efficient match [16,17], mate preferences can be complex. Research has shown that choosers can select a broad range of phenotypes including signals outside the range of natural conspecific variation, such as novel signals, through the expression of latent preferences [18,19] (e.g. spiders [20], grasshoppers [21] and guppies [22]). In some cases, mate preferences can favour heterospecifics [23].

The jumping spider genus *Habronattus* is a compelling system in which to study hybridization and mate choice. *Habronattus* is a specious group (>100 species) with evidence that a majority of species diversified in the past 2.5 Myr [24]. Some studies have suggested that female choice plays an important role in diversification [25,26]. Male courtship displays vary across the genus, from simple to elaborate displays involving different ornamentation, movement, vibratory and tactile signals [25,27]. Despite elaborate displays, male *Habronattus* frequently target their courtship to heterospecifics [28,29]. Additionally, despite trichromatic vision [30] and behavioural evidence that females can detect and assess vibratory signals [31], there is evidence for incomplete assortative mating between species [20,26,32], ancient and recent introgression [33–36] and multiple hybrid zones [37,38]. Distinct male courtship displays with frequent gene flow across species boundaries provide a unique opportunity to test how mate choice patterns influence patterns of hybridization that can lead to genomic homogenization on one hand and diversification (i.e. adaptive introgression [39] and hybrid speciation [1]) on the other.

Among *Habronattus*, the *americanus* species complex is of interest because mate choice is likely important in determining the likelihood of hybridization owing to post-zygotic isolation being weak [38]. Members of the *americanus* subgroup have relatively simple dance and vibratory displays but marked variation in coloration [36]. While morphological data suggest that several species exist with geographical variation, genomic data suggest that genetic groupings are consistent with geography and not morphology [36]. Hybrid zone studies between *H. americanus* whose parental phenotype consists of red males and *Habronattus kubai* whose parental phenotype consists of brown and black males revealed a range of intermediate and transgressive phenotypes dominated by red individuals (*H. americanus* phenotype) [38]. These studies suggest the possibility that morphological and genomic data are discordant because mating preferences for red coloration are driving genomic homogenization in the *americanus* subgroup. Consistent with this, the ‘sea of red’ hypothesis posited by Bougie *et al.* [38] suggests that preferences for red phenotypes explain species distributions where taxa other than *H. americanus* are being replaced by *H. americanus* (‘extinction via hybridization’; [13–15]).

To test predictions from the ‘sea of red’ hypothesis and how mate preferences could lead to extinction via hybridization, we selected two allopatric populations [36] representing the extremes of coloration variation in the *americanus* subgroup. *Habronattus americanus* PLC-form males show red ornamentation on the anterior surface of the pedipalps and chelicerae, as well as on the anterior ventrolateral side of the first femur. On the other hand, *Habronattus sansoni* CC-form males are brown overall with two black stripes above their principal eyes. Both populations perform vibratory displays including percussive and tremulatory elements. We first conducted con- and heterospecific mating trials (Experiment I) to test predictions from the ‘sea of red’ hypothesis [38]; if *americanus* subgroup females are biased towards red coloration, we predicted that both *H. sansoni* CC and *H. americanus* females would prefer to mate with red *H. americanus* PLC males. Next, to test whether red coloration drives the observed mating patterns, we manipulated male colour in conspecific pairs (Experiment II). If red coloration is responsible, we predicted that red-coloured males would be preferred over brown-coloured males in both species. Finally, we visualized the distribution of *H. americanus* and *H. sansoni* colour morphs using iNaturalist observations to examine whether colour morph distributions were consistent with the ‘sea of red’ hypothesis and the predictions drawn from our behavioural experiments (Experiments I and II). Overall, mate choice preferences are key to understanding the phylogenetic, geographical and morphological relationships found in the *americanus* species group and provide insights into the role of latent preferences in speciation dynamics.

## 2. Methods

### (a) Animal collection

We collected *H. sansoni* (CC morph: mostly brown with black stripes above principal eyes) from near Cedar City, Utah, from late May to early June 2023. The spiders are found on aspen and/or conifer litter on exposed ground. *Habronattus americanus* (PLC morph: red in pedipalp, leg and chelicera) [26] were collected from South Lake Tahoe, CA in mid-June of 2023. The spiders are found on conifer litter in exposed areas near sagebrush (*Artemisia*). For animal care procedures, see electronic supplementary material.

### (b) Courtship displays

To characterize courtship displays, we recorded male displays to a female model following standardized procedures [40]. Representative video vouchers of courtship displays are archived at the Macaulay Library of Natural Sounds (*H*. *americanus* PLC: ML 488859; *H. sansoni* CC: ML488860). For details, see electronic supplementary material.

### (c) Experiment I: heterospecific mate choice trials

To explore whether red-ornamented *H. americanus* PLC males are preferred by *H. sansoni* females, we conducted heterospecific mate choice trials in a 2 × 2 design (N♀amer♂amer = 44, N♀amer♂sans = 38, N♀sans♂amer = 31 and N♀sans♂sans = 35). For each mating trial, we placed the female in the arena and allowed her to acclimate for 5 min. We then placed the male into the arena and allowed them to freely interact for 15 min. We ended mating trials before 15 min when there was copulation or cannibalism. After a mating trial, we measured body mass (Ohaus Analytical Plus Laboratory Scale, Wazobia Enterprise). The average age of *H. sansoni* females was 23.79 ± 2.64 (mean ± s.d.) days post maturity and *H. americanus* females 24.32 ± 3.04 (mean ± s.d.) days post maturity.

Using Behavioral Observation Research Interactive Software (BORIS, Olivier Friard), we measured the duration of the visual phase (see below), multimodal phase (see below) and whether a male mounted a female (copulation). For the visual phase, the behaviour started when the male oriented towards the female and raised both anterior legs and ended when the male either initiated the multimodal phase or stopped displaying. For the multimodal phase, the behaviour started when the male began to move its anterior forelegs while producing a vibratory signal and ended when the male attempted to mount the female, transitioned to the visual phase, was chased away by the female, or stopped courting.

**Figure 1.**
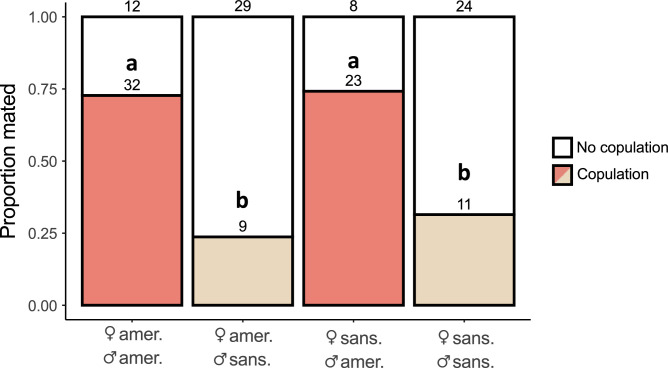
*Habronattus americanus* PLC males are preferred by females of both species. Bar plot representing general courtship outcome. Female and male species are labelled on the *x*-axis: ♂amer.: *H. americanus* PLC male, ♂sans.: *Habronattus sansoni* CC male, ♀amer.: *H. americanus* female, ♀sans.: *H. sansoni* female. Coloured bars (red and brown) indicate mated pairs and white bars indicate pairs that failed to copulate. Letters indicate statistical significance (chi-squared tests).

**Figure 2.**
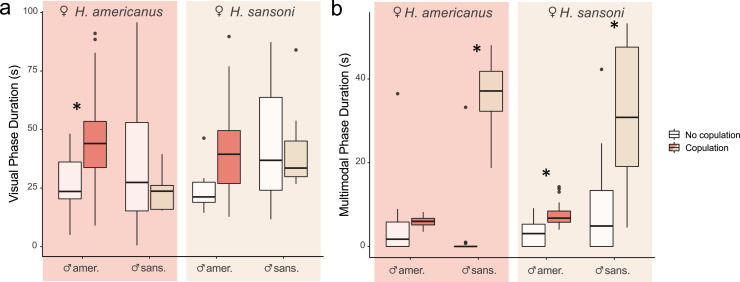
The visual and multimodal courtship phases play different roles in courtship outcomes. Male species are labelled on the *x*-axis: ♂amer.: *Habronattus americanus* PLC male, ♂sans.: *Habronattus sansoni* CC male. Female species are labelled at the top of the coloured boxes. Coloured boxes (red and brown) indicate mated pairs and white boxes indicate pairs that failed to copulate. Asterisks indicate statistical significance (boostrapping, see electronic supplementary material, figure S3). (a) Boxplot comparing mean visual phase duration of mated and unmated males in each pairing group. (b) Boxplot comparing mean multimodal phase duration of mated and unmated males.

To compare the mating rate between hetero- and conspecific pairs (figure 1), we performed a chi-squared test using the R function chisq.test() in the *stats* package. To explore the role that courtship duration plays in mating success in heterospecific and conspecific matings (figure 2), we used a non-parametric method by bootstrapping each subset of data stratified by copulation and pairs (AA, AS, SA and SS) and calculating the mean. This process was repeated 1000 times and statistical significance was determined according to whether the 95% confidence interval overlapped for individuals that did and did not copulate [41]. We did not use conventional parametric methods because model assumptions were not met after data transformation. We did not use conventional non-parametric methods because they do not specifically draw conclusions on the mean of the subsets [42–44]. Details on the statistical significance of the bootstrapping test can be found in the electronic supplementary material, figure S3.

**Figure 3.**
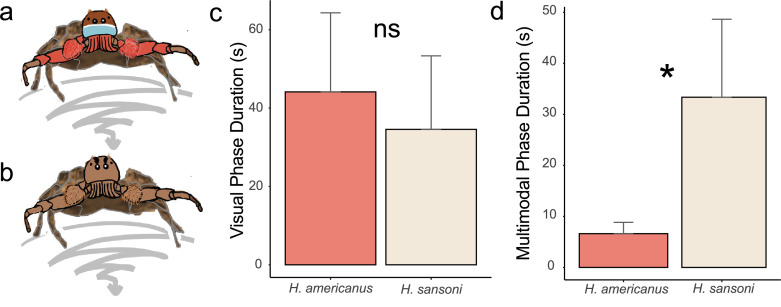
Visual phases of *Habronattus americanus *PLC and *Habronattus sansoni *CC courtship do not differ in duration, but *H. sansoni* CC engage in longer multimodal phases. (a,b): Visual phase display posture where males of both species (a: *Habronattus americanus* and b: *Habronattus sansoni*) zigzag (grey paths) towards the female. (c) Comparison of mean visual phase duration between the two species. (d) Comparison of mean multimodal phase duration between the two species. Error bars in both cases represent standard deviations.

For each species, male courtship in con- and heterospecific trials did not differ (electronic supplementary material, figure S3) thus we combined species data (figure 3). To compare the duration of the visual and multimodal phases of *H. americanus* PLC and *H. sansoni* CC males used in our experiments, we built linear regression models using data from all trials in which the male successfully mated, with male species as a binary predictor and phase duration as the response variable. Only males that successfully mated were used in this analysis since those that did not copulate did not perform the full display and are thus not representative of a ‘complete’ courtship display. We removed 4 outlier trials with long visual courtship phases (>100 s) and another 8 outlier trials with long multimodal courtship phases (>56.25 s). All the outlier trials removed involved *H. sansoni* CC males. Note that the inclusion of these trials in the models did not change the patterns observed (see below). After checking for model assumptions, we performed a type II analysis of variance (ANOVA) to determine statistical significance using the function Anova() in the *car* package [45]. All statistical analyses were performed in R v. 4.1.3 [46]. See electronic supplementary material for details on arena set-up.

### (d) Experiment II: conspecific colour manipulation trials

We randomly assigned 38 *H*. *sansoni* CC and 45 *H*. *americanus* PLC males into two groups: red (experimental) and brown (control). Twenty *H. sansoni* CC were designated for the control treatment and 18 for the experimental treatment. Twenty-three *H. americanus* PLC were designated for the control treatment and 22 were designated for the experimental treatment. For the colour manipulation, we anesthetized spiders under a steady stream of 0.068 l min^−1^ CO_2_ (1 psi). Next, using a paint brush, we placed the spider into a styrofoam cut-out (1.5 cm × 1.5 cm × 2.5 cm). Under a dissection microscope (Leica M80 Stereo Zoom Microscope, Leica Biosystems), we painted over the anterior surface of the pedipalps, chelicerae and ventrolateral side of the first leg femur [26,36] with either red (Liquid Eyeliner Red, Handaiyan Eyeliner color01) or brown waterproof eyeliner (Liquid Eyeliner Brown, Handaiyan Eyeliner color10).

After the procedure, we waited 5 days for males to recover and for any volatile compounds to dissipate. Before the start of each trial, males were evaluated visually to ensure that colour treatments persisted. Afterwards, each male was paired with a conspecific female. All mating trials were run as described above. The substrate was changed after each mating trial to remove any chemical cues. The average age of *H. americanus* females was 70.04 ± 22.40 (mean ± s.d.) days post maturity and *H. sansoni* females 67.5 ± 18.98 (mean ± s.d.) days post maturity.

**Figure 4.**
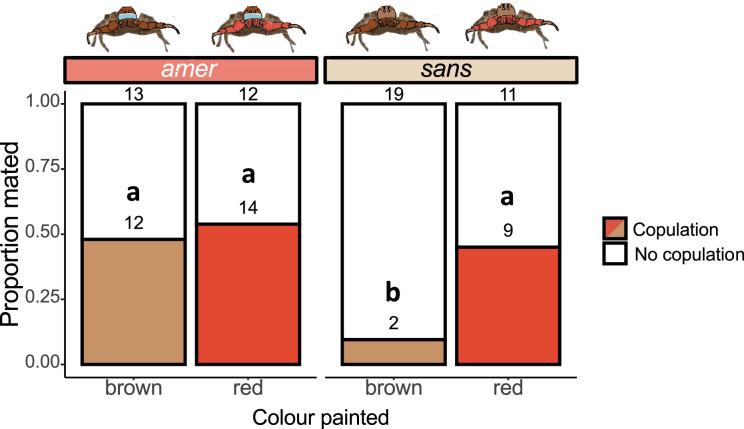
Colour manipulations did not alter mating outcomes in *Habronattus americanus* pairings but altered mating outcomes in *Habronattus sansoni* CC pairings where females preferred red-painted males. Illustrations of colour-manipulated males are above the horizontal bars with species identities labelled in the horizontal bars above the bar plot. For the bar plot, coloured bars (red and brown) denote successful copulation and white bars denote pairs that failed to copulate. Sample sizes of each treatment are provided at the top of each bar. Letters indicate statistical significance (chi-squared, p<0.05).

To compare the mating rate between treatments, we performed a chi-squared test using the R function chisq.test() in the *stats* package (figure 4). To explore the role of courtship duration in mating success for our treatments (electronic supplementary material, figure S2), we used a non-parametric method by bootstrapping each subset of data stratified by copulation and treatment and calculating the mean. This process was repeated 1000 times, and statistical significance was determined by whether the 95% confidence interval overlapped for individuals that did and did not copulate [41]. We did not use conventional parametric or non-parametric methods for the same reasons as described above in Experiment I. Details on the statistical significance of the bootstrapping test can be found in the electronic supplementary material figure S3. All statistical analyses were performed in R v. 4.1.3 (46).

### (e) The geographical distribution of *Habronattus americanus* and *Habronattus sansoni* colour morphs

Research grade observations of adult male *H. americanus* and *H. sansoni* from iNaturalist (https://www.inaturalist.org/) were compiled with species, coordinates and accuracy of coordinates. Note that all *H. americanus* and *H. sansoni* morphs are included in this analysis. There are nine informally described *H. americanus* morphs that vary in red ornamentation [36], including one morph that has no red coloration [47]. There are currently three informally described *H. sansoni* morphs that vary in face ornamentation and foreleg ornamentation, including one morph that has red facial stripes [36]. The time range of iNaturalist observations included in this study is from May 2013 to July 2024. The presence/absence of red coloration is based on posted images. Observations where coloration could not be evaluated were discarded. We further discarded data points with a resolution lower than 1000 m.

To visualize geographical distributions, we compiled elevation data using the *elevatr* package in R [48]. The zoom level for this procedure was set to 6. All elevations below sea level were set to NA to enhance the contrast of the visualization. The state borders were acquired from the *rnaturalearth* package [49] and plotted using *ggplot2*.

## 3. Results

### (a) Courtship display descriptions

*Americanus* subgroup displays can be divided into two phases: (i) visual phase and (ii) multimodal phase that consist of stereotyped visual (i.e. ornaments, motion and posture) and vibratory displays (electronic supplementary material, video). Visual display phases of *H. americanus* PLC and *H. sansoni* CC occur as the male approaches in a zigzag motion, shifting his body laterally from one side to another [50]. The multimodal phase begins when the male stops moving (see above) and begins to move his forelegs back and forth, producing a vibratory multicomponent signal [50]. Two vibratory signal components are produced during this phase (electronic supplementary material, figure S1), (i) a buzzing sound that continues throughout the multimodal phase and (ii) a flicking sound that aligns with the back-and-forth movement of the forelegs. Both vibratory components form a multicomponent vibratory signal [51]. While the motion and postural elements of the multimodal phase are qualitatively similar between species, the vibratory elements differ temporally. *Habronattus sansoni* CC males perform the multicomponent vibratory signal more slowly but for a longer duration, while *H. americanus* PLC males perform the multicomponent vibratory signal more quickly but for a much shorter length of time. For the purposes of this study, we quantify differences between displays for each species using data from Experiment I (see below). See electronic supplementary information for more details on courtship displays.

### (b) Experiment I: heterospecific mate choice trials

*Habronattus americanus* PLC males are preferred by females of both species (figure 1). For *H. americanus* females, the mating rate when presented a *H. sansoni* CC male is 24% (9/38 mated), while the mating rate when presented a *H. americanus* PLC is 73% (32/44 mated)(*χ*^2^ = 17.705, d.f. = 1, *p* < 0.001, Pearson’s chi-squared test). This pattern is similar for *H. sansoni* CC females; the mating rate when presented a *H. sansoni* CC male is 31% (11/35 mated), while the mating rate when presented a *H. americanus* PLC male is 74% (23/31 mated) (*χ*^2^ = 10.386, d.f. = 1, *p* = 0.001, Pearson’s chi-squared test).

*Habronattus americanus* PLC males display red ornamentation on their pedipalps, forelegs and chelicerae (figure 3a). Although *H. sansoni* CC males have a similar visual phase of courtship, they do not possess red ornamentation as they are entirely brown and black (figure 3b). For individuals used in this study, the mean duration of the visual phases of both species is similar (*H. americanus *PLC: 44.16 ± 20.16 s, mean ± s.d., *n* = 55; *H. sansoni* CC: 34.58 ± 18.76 s, mean ± s.d., *n* = 12; *F-*value = 2.3, d.f. = 1, *p* = 0.14) (figure 3c). On the other hand, *H. sansoni* CC males (mean ± s.d. = 33.35 ± 15.28 s, *n* = 12) perform significantly longer multimodal displays than *H. americanus* PLC males (mean ± s.d. = 6.62 ± 2.22 s, *n* = 55) (*F-*value = 172.7, d.f. = 1, *p* < 0.001) (figure 3d).

Males that successfully copulated in the the ♀A♂A cross (copulation: mean ± s.d. = 46.09 ± 20.26 s; no copulation: 27.12 ± 14.39 s) had longer visual phase durations but this was not true of the other crosses (♀A♂S, ♀S♂A, ♀S♂S) (figure 2a). Note that in the ♀S♂A pair, there is a hint that males that copulated demonstrated longer visual phases (figure 2a), but this was not statistically significant using our conservative approach (electronic supplementary material, figure S3). Males that copulated in the ♀A♂S, ♀S♂S and ♀S♂A crosses, but not the ♀A♂A cross, demonstrated a longer multimodal display duration (figure 2b).

### (c) Experiment II: conspecific colour manipulations trials

If red coloration is a major factor that determines mating, we predicted that red-painted *H. sansoni* CC males would have a higher mating rate than their brown-painted (control) counterparts, and brown-painted *H. americanus* PLC males would have a lower mating rate than their red-painted (control) counterparts. Our results show that red *H. sansoni* CC males have significantly higher mating rates than control conspecifics (45% versus 11% mated, *χ*^2^ = 4.885, d.f. = 1, *p* = 0.027, Pearson’s chi-squared test) (figure 4). However, the mating rate of *H. americanus* PLC did not differ significantly between red- or brown-painted males (54% versus 48% mated, *χ*^2^ = 0.019, d.f. = 1, *p* = 0.891, Pearson’s chi-squared test) (figure 4). The duration of the visual phase did not differ between pairs that did or did not copulate, but almost every male that proceeded to the multimodal courtship phase mated (electronic supplementary material, figure S2). For the control (brown-painted) *H. sansoni* CC group, pairs that copulated (mean ± s.d. = 63.06 ± 0.05 s) had a longer visual phase than those that did not copulate (25.52 ± 16.27 s) (electronic supplementary material, figure S2a). In the case of the multimodal phase, it is similar to the result in *H. americanus* in that nearly all males that progressed to that stage copulated (electronic supplementary material, figure S2b). We note that the number of brown-painted *sansoni* males that mated is low (2/21, figure 4); thus, the results from this comparison should be considered preliminary. We also note that while our results show the influence of red coloration, we cannot distinguish whether these results stem from perceived changes in hue, chroma, brightness, chromatic contrast, achromatic contrast or a combination of these features.

### (d) The geographical distribution of *Habronattus americanus* and *Habronattus sansoni* populations

**Figure 5.**
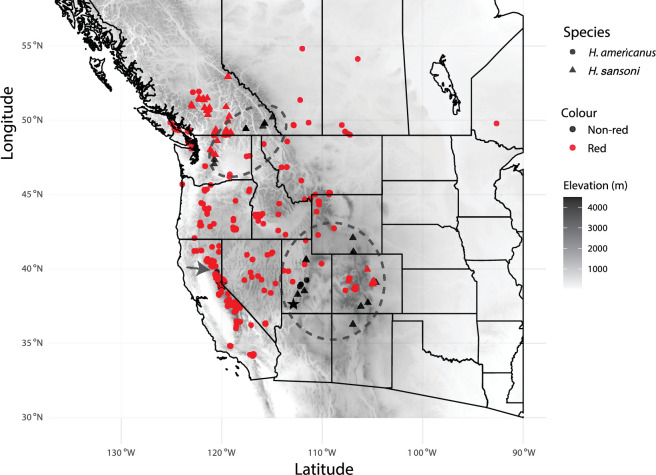
Red coloration dominates populations of *Habronattus americanus* and *Habronattus sansoni*. Grey shading in the map indicates elevation and state borders are shown by black solid lines. Red points indicate observed males with red coloration, and black points indicate males without any red coloration. Circle points indicate *H. americanus* males and triangle points indicate *H. sansoni* males. The *H. americanus* PLC and *H. sansoni* CC used in this study are marked on the map with red and black stars, respectively. Two dashed black circles indicate pockets where non-red males were observed. The black arrow points to the Portola population of non-red males.

Geographical distributions of *H. americanus* and *H. sansoni* populations from iNaturalist data suggest that the red coloration dominates across the distribution of both species (figure 5). Non-red coloration appears in three pockets (figure 5). The geographical distribution of red coloration is consistent with the ‘sea of red’ hypothesis [38].

## 4. Discussion

In this study, we investigated mating in the *H. americanus* subgroup using an experimental design that simulated secondary contact between allopatric populations of *H. americanus* and *H. sansoni*. One primary goal was to understand the behavioural patterns that may be driving diversification patterns observed in this species complex [34–36]. We found that the displays of *H. americanus* PLC and *H. sansoni* CC differed in some respects (ornamentation colour and multimodal phase) but not in others (visual phase). For conspecific *H. americanus* crosses, successful males had longer visual phases compared with unsuccessful males, and successful males had longer multimodal phases for both con- and heterospecific *H. sansoni* CC crosses as well as for heterospecific *H. americanus* PLC crosses. Interestingly, our results showed that females of both species preferred *H. americanus* PLC males. To test whether red coloration drove the observed mate choice patterns, we conducted a colour manipulation experiment and found that colouring *H. sansoni* CC males red significantly increased their mating success, while colouring *H. americanus* PLC males brown had no effect. Together these results suggest strong latent preferences for red coloration in *H. sansoni* CC and that secondary contact could lead to hybridization and introgression with a biased directionality (*H. sansoni* to *H. americanus*), consistent with the ‘sea of red’ hypothesis [38]. On the other hand, our results suggest weak to no preferences for red coloration in *H. americanus* and instead, that mate choice in *H. americanus* is based on a more complex combination of visual (posture and colour) and vibratory information.

### (a) Signal function and signalling trade-offs

In spiders, sexual signals may provide information about mate quality and species identity, attract attention, prevent aggression and/or stimulate females to mate [52]. Visual phases may function to attract attention [53] by contrasting with the background to potentially prevent aggression [54–56]. Our results suggest that the visual phase is particularly important for *H. americanus*. It is possible that red ornaments in *H. americanus* function to help them to stand out from the brown background of the forest floor [57,58]. While this may be the case, all mate choice trials in this experiment were conducted in the same uniform environment. One possibility is that red coloration might be an ‘aesthetic’ preference providing no other function than being attractive [59,60]. In heterospecific crosses, this may be an example of a hidden aesthetic preference (red coloration in *H. americanus* PLC males) [61] overriding existing preferences [62] (longer multimodal phase in *H. sansoni* CC males; see discussion below).

Multimodal phases may also function to provide information about mate quality and species, attract attention, prevent aggression and/or function to overload the sensory system of females [52,53,63,64]. Our data indicate that multimodal phases are likely important for both *H. americanus* PLC and *H. sansoni* CC. In *H. sansoni* CC conspecific trials, females prefer males that produce longer multimodal courtship. Longer courtship duration, especially multimodal courtship, has been shown to predict mating success in various groups [65,66], including jumping spiders [67], potentially as indicator traits of fitness owing to the high metabolic cost of acoustic signals [53,68]. Interestingly, although *H. sansoni* CC males emphasize this aspect of their display and females prefer longer multimodal displays, the presence of red overrides this preference. This is further supported by our findings that red-painted *H. sansoni* CC are more likely to mate than brown-painted *H. sansoni* CC. Our results suggest a trade-off between the duration of multimodal courtship and red coloration, with *H. sansoni* CC males spending a significantly larger amount of time performing multimodal courtship. This may indicate cost-based trade-offs. While our study focuses on the role of female preferences, males may also assess cues of female receptivity and respond accordingly. Currently, we cannot distinguish the contributions of male assessment.

### (b) Function of red coloration

Red coloration has been shown to play an important role in sexual selection in many animal systems such as guppies, house finches, sticklebacks and lizards [69–71] as well as jumping spiders [31,72,73]. Preferences for red coloration may be driven by mechanisms such as perceptual/sensory bias [74,75] and its links to mate quality [76,77]. Preferences for red coloration could also arise from enhanced contrast (achromatic and chromatic) with the signalling environment [56,57,78], even in animals that cannot perceive red [56,78]. Red is also associated with important food items in a wide range of taxa such as fish [74], birds [79–81] and primates [82] and it may be a signal of food-gathering ability (i.e. carotenoid-based coloration). The acquisition of carotenoids and its links to, among other things, parental care [83,84], body condition [85] and immune competence [86,87] have been posited as explaining the use of red ornaments in a variety of taxa. However, the fact that *H. sansoni* CC females prefer a red trait that does not exist in conspecific males suggests that it arose through a perceptual bias for red. Further experiments that examine females’ response to red coloration outside of mating contexts (e.g. predation) are necessary to test this hypothesis.

Red sexual ornaments have been described in several species of jumping spiders. Interestingly, despite its prevalence in many groups, evidence for the use of red coloration in mate choice has been weak. Red only improves mating success under sunlight in *Habronattus pyrrithrix* [31,88]; *Saitis barbipes* lacks red photoreceptors necessary to perceive the red contained in the red ornaments of males [78]; contrast seems to matter more than red coloration *per se* in *Maratus volans* [56]; and male *Lyssomanes viridis* with more red in their chelicerae tend to lose fights [72]. While many salticids are dichromatic [89], *Habronattus* spiders are able to see red using a retinal filter [30] and red coloration is common in males of many *Habronattus* species (e.g. *H. americanus, H. pyrrithrix, H. virgulatus* and *H. coecatus* etc.). The presence of red secondary sexual characters and their position on body parts that are displayed to females suggest their function in courtship. Interestingly, many *Habronattus* male juveniles also have a red-coloured stripe below the principal eyes (= clypeus), which in most species disappears at maturity [90]. The presence of red across *Habronattus* species suggests that the salience of red coloration has driven the evolution of ornaments (sexual or otherwise) multiple times.

Our study supports the hypothesis that a general preference for red coloration in males exists in the *H. americanus* subgroup. A mutual preference for red coloration in females across the *americanus* subgroup would predict that red ornamentation would dominate in any population where the trait is present, and that over multiple generations of hybridization and introgression, populations lacking red would eventually be taken over by populations with red males. The current distribution of *H. americanus* and *H. sansoni* populations supports this pattern, with the majority of the distribution of *H. americanus* and *H. sansoni* consisting of males with red ornamentation (figure 5). Geographical distribution maps suggest that the few non-red populations of *H. americanus* and *H. sansoni* group into three ‘islands’, one located in Utah, Colorado and southern Wyoming, one in northern Washington and neighbouring British Columbia and a smaller pocket located in northeastern California. As posited by the ‘sea of red’ hypothesis [38], it is as if populations with red secondary sexual traits flood the landscape, and non-red populations are the last isolated islands in this sea of red.

### (c) Implications for speciation dynamics

In the literature, the behavioural mechanisms driving hybridization, particularly widespread hybridization such as that observed in the *americanus* subgroup [2,36], have been attributed to ‘errors’ in mate choice driven by habitat degradation (particularly anthropogenic disturbances) and its effects on the ability to distinguish between species [91,92]. Our results point to an alternative mechanism for widespread hybridization based on a strong preference that is outside the range of conspecific variation. Mate preferences outside the range of natural variation, as shown in the current study, may be the result of (i) permissive or (ii) latent preferences. Permissive preferences refer to a ‘wide’ fitness peak in a preference function. For example, female Hawaiian crickets are thought to select variants of male songs because females will approach male signals with a wide variety of frequencies [93]. While permissive preferences emphasize the range of preference functions as they relate to existing trait variation, latent preferences emphasize preference for traits that are not expressed. Latent preferences occur owing to ‘hidden’ fitness peaks in preference functions that are not explored because courters do not express those traits. Perceptual biases and neophilia fall under this framework [22,94,95]. Perceptual biases in the context of mate choice refer to biases for certain features that evolved outside reproductive contexts (e.g. foraging and predation) or for features that more effectively stimulate an organism’s sensory systems [75]. Neophilia is a preference for a trait that is unfamiliar/novel. Compared with perceptual biases that emphasize the trait and the perceptual system of the chooser, neophilia emphasizes escape from habituation [22]. Given the major differences between coloration patterns, we suggest that *H. sansoni* CC preferences for red represent a latent preference. With our design, we cannot disentangle whether the preference for red in *H. sansoni* females is driven by perceptual bias or neophilia. Further experiments with additional colour manipulations can provide more insights. Based on the widespread presence of red coloration in males in *Habronattus*, including in non-sexual traits [90], it is more likely that perceptual biases drive the pattern observed.

One surprising result from our study is that painting *H. americanus* brown did not affect its likelihood of mating. These results suggest the intriguing possibility that once expressed and spread in a population/species, strong preferences for red can become relaxed. The same pattern was also found in Poeciliid fishes [96]. In this example, preference for swordtails is found in female platyfish although male platyfish are swordless [97]. Preference for swords was found to be weaker in swordtails compared with swordless platyfish [97]. Reduced selection makes it possible for choosers to select mates according to a combination of traits as opposed to one strongly preferred trait. Intriguingly, looking across different *H. americanus* populations, the variation in the use of red ornaments is much greater than the colour variation seen in any of the other *americanus* subgroup members. In two populations of *H. americanus,* red ornamentation appears to be nearly absent [47]. The disappearance of strong preferences for red may be the result of antagonistic coevolution similar to arguments posited for explaining the xenophilic/neophilic preferences in the *Habronattus pugillis* group [20,32]. In those studies, females of some populations (Santa Rita Mountains, AZ) preferred males that used vibratory signals (Atascosa Mountains, AZ) not expressed in their own local populations of males [20]. In this scenario, males evolve novel exploitative signals and females subsequently evolve resistance to males from their own populations. However, they do not develop resistance to novel traits expressed in other populations to which they have not been exposed to but that share their perceptual biases. Similar to this study, manipulating signals produced by local males did not affect mating success, while manipulating the signals by the non-local populations eliminated the preference for non-local males [20]. Another possibility is that predation pressures are higher on red individuals, leading to a weakened preference for red assuming some genetic linkage between the red trait and preference for red. This hypothesis was posited as explaining reduced preferences for swords in swordtail fish [98]. Alternatively, the red coloration of *H. americanus* is indeed important for female choice, but our choice of red colour masked the natural coloration that females strongly preferred. It is important to note that the females used in the conspecific colour manipulation experiment were older than those used in the heterospecific mate choice experiment. It is possible that some of our results may reflect shifts in female preferences as they age, explaining, for example the differences that we observed in mating rates between the two experiments. Given these potential differences, we caution against directly comparing results from the heterospecific mating trials (Experiment I) with the conspecific colour manipulation trials (Experiment II). Future work in the *americanus* subgroup that manipulates females’ ability to assess coloration and explores age-related changes to mate choice is necessary to explore these possibilities.

Nevertheless, our results suggest that strong preferences for novel traits can override existing preferences for traits expressed by conspecifics and will likely have strong impacts on the evolutionary trajectory of the populations in question. Two previous studies point to two possible evolutionary trajectories. First, Bougie *et al.* [38] documented an existing hybrid zone between *H. americanus* and *Habronattus kubai*, which may point to what may happen in a timeline where two populations—one with a strong, pre-existing preference for a trait exhibited by the other—come into secondary contact. In this study, Bougie *et al.* documented a genetically homogeneous ‘hybrid swarm’ with more hybrid than parental phenotypes but where red phenotypes of one population (*H. americanus*) occurred at much higher frequency than the phenotype of the other (*H. kubai*). Furthermore, this study suggested that non-red phenotypes were localized to microhabitats (beneath shrubs) that were shadier [38], consistent with findings that the processing of red is only possible in bright light environments [31]. This study exemplifies a scenario where a strong preference from one population drives widespread directional introgression, potentially resulting in a period of lineage ‘fusion’ and the possible extinction of *H. kubai* (extinction by hybridization [99]).

Our manipulative experiment however points to a scenario where this strong preference is ephemeral because painting *H. americanus* brown had no effect on mating. In other words, the presence of red coloration in males weakens the preference for red, thus making the strong preference for red transient. This relaxed selection for red males might give opportunities for other colour morphs to evolve. The evolutionary trajectory emerging from this may be exemplified by a study by Blackburn & Maddison [26], who documented widespread gene flow between different morphs of *H. americanus*. Blackburn & Maddison [26] documented phenotypically divergent populations of *H. americanus* (three morphs) in a small area with high genetic similarity but with evidence for isolation by distance likely caused by drift. This study would exemplify a scenario where a strong preference for a simple trait has been weakened, potentially through the evolution of female habituation [100–102] or counterbalancing natural selection pressures leading to the evolution of a variety of secondary traits, as seen across *H. americanus* males [26,36]. Female habituation and preference for novel traits have been shown to drive genetic diversity in guppies [22], and the same mechanism might have given rise to the ornament diversity in the *americanus* subgroup. For these females, preferences would be based on the evaluation of multiple signals, as suggested by our conspecific *H. americanus* mating data. This may signal a ‘fission’ epoch in speciation dynamics [103]. Importantly for this study, unlike adaptive radiations such as cichlid fish in the African Rift Lakes, Darwin’s Finches in the Galapagos and Hawaiian Silverswords, diversification in most *Habronattus* species groups is likely not driven by ecological processes (but see [33]). The *Habronattus* system may illuminate diversification dynamics driven primarily by sexual selection, as suggested by previous authors [25]. It is important to note that while there is no direct evidence of a current hybrid zone between *H. americanus* and *H. sansoni*, genomic evidence suggests that they have interbred in the past given the limited genomic differentiation between all members of the *americanus* subgroup [34,36]. Additionally, the existence of several hybrid zones for other *americanus* subgroup members [38] suggests that hybridization in the subgroup produces viable and fertile offspring. Future work is needed to verify this hypothesis.

## 5. Conclusion

The present study suggests that perceptual biases could be a mechanism that leads to hybridization among closely related species. Furthermore, our study suggests that some preferences may be so strong as to override existing mate choice patterns and in extreme situations drive lineage fusion. This phenomenon, however, could be ephemeral and may provide opportunities for novel traits (and potentially species) to evolve through adaptive introgression [104–108] and the enhanced evolvability of hybrids [109]. Similar to the spectacular diversifications of African Cichlids and Hawaiian Silverswords that had their roots in ancient hybridization events [99,103,110,111], extensive cyclical hybridization in *Habronattus* [34] may be an important contributor to rapid diversification and the spectacular diversity of displays found across the genus [27,32,38,77].

## Data Availability

Data and code accessible at: [112]. Supplementary video: [113]. Supplementary material is available online [114].
